# A generalized heat conduction model of higher-order time derivatives and three-phase-lags for non-simple thermoelastic materials

**DOI:** 10.1038/s41598-020-70388-1

**Published:** 2020-08-12

**Authors:** Ahmed E. Abouelregal, K. M. Khalil, F. A. Mohammed, M. E. Nasr, Adam Zakaria, Ibrahim-Elkhalil Ahmed

**Affiliations:** 1 Department of Mathematics, College of Science and Arts, Jouf University, Al-Qurayyat, Saudi Arabia; 2 Department of Mathematics, Faculty of Science, Mansoura University, Mansoura, 35516 Egypt; 3 Mathematics Department, Faculty of Science, Benha University, P.O. 13518, Benha, Egypt; 4 Department of Mathematics, South Valley University, Qena, Egypt; 5 Department of Mathematics, Faculty of Science, University of Kordofan, El-Obeid, Sudan; 6 Departement of Mathematics, Faculty of Science and Technologe, Shendi University, Shendi, Sudan

**Keywords:** Materials science, Mathematics and computing

## Abstract

In the current work, a new generalized model of heat conduction has been constructed taking into account the influence of the microscopic structure into the on non-simple thermoelastic materials. The new model was established on the basis of the system of equations that includes three-phase lags of higher-order and two different temperatures, namely thermodynamic and conductive temperature. The two-temperature thermoelastic model presented by Chen and Gurtin (Z Angew Math Phys 19(4):614–627, 1968) and some other previous models have been introduced as special cases from the proposed model. As an application of the new model, we studied the thermoelastic interactions resulting from sudden heating in an isotropic solid subjected to external body force. The influence of the discrepancy parameter and higher-order of the time-derivative has been discussed. This work will enable future investigators to gain insight into non-simple thermoelasticity with different phase delays of higher-order in detail.

## Introduction

Duhamel (1837) was the first to suggest entering the coupling term in the heat equation and proposing the coupling between the temperature and the deformation. However, the heat equation was not well established by the thermodynamic process. Biot^1^ attempted the thermodynamic justification for this equation. The heat equation includes, in this case, the dilatation term depend on the thermodynamics of the non-inverse process. The classical heat conduction theory in thermoelastic solids is based on the assumption that the heat flow is proportional to the temperature gradient. Based on this assumption, the heat equation is governed by a parabolic system of partial differential equations, which predicts that the thermal disturbance in the material will immediately affect all points of the body. The phenomenon of infinite velocity of heat waves conflicts with physical phenomena.

To overcome this defect, generalized thermodynamic theories were introduced. Lord and Shulman^2^ introduced a generalized theory of thermoelasticity that provides one relaxation time and thus the system that governs the heat equation has turned into a hyperbolic type. Green and Lindsay^3^ proposed another model involved two relaxation times.

The following generalization of the theory of thermoelasticity is known as the dual-phase-delay model which was improved by Tzou^3^ and Chandrasekharaiah^4^. Tzou^3^ reflected a constitutive equation to explain the lagging behavior in the heat conduction in solid materials. Tzou^5,6^ presented a thermoelastic model including dual-phase-delay to the heat flux vector and the temperature gradient.

The two-temperature theory (2TTE), proposed by Chen and Gurtin^7^, Gurtin and Williams^8,9^ and Chen et al.^10,11^, based on two distinct temperatures; conductive $\varphi$ and the thermodynamic $\theta$ temperatures. According to this theory, the difference between these two temperatures is proportional to the applied heat source. Also, in the absence of the heat source, the two temperatures are equal^12^. Based on this theory of thermoelasticity with two-temperature, several authors have studied several problems of thermoelasticity^13–18^. Quintanilla^19^ discussed the structural stability, existence, and spatial behavior of the solution of some problems in 2TT. For isotropic and homogeneous bodies, Youssef^20^ introduced the theory of thermoelasticity with relaxation time and two-temperature. Mukhopadhyay et al.^21^ also, extended generalized thermoelasticity with two temperatures and dual-phase-lag. The two temperatures theory has attracted a lot of attention in the recent years^22–26^.

Recently, many efforts have been introduced to modify the classical heat conduction law. In one of these efforts, Abouelregal^27–29^ introduced some generalized models of heat conduction including higher-order time-derivative. Earlier, Chiriţă^30^ investigated the high-order Fourier law to illustrate the lagging performance of heat transfer.

The current paper is concerned with a generalized model that combines the two-temperature theory and the heat conduction of higher-order time-derivatives with two-phase-lags. To further study the accuracy of the current model, the model was applied to study the interaction in an isotropic solid exposed to external body force and due to sudden heating. Some special cases of concern were also deduced from the proposed model. For further clarification and comparison, the numerical results were tabulated and illustrated graphically. The effects of the parameters of temperature distinction, phase delay, and high-orders on all field variables inside the material have been studied. The numerical results obtained in this paper were found to be as good as with the results in the current literature. Also, the results and observations also showed that the analytical solutions correspond well with numerical solutions.

## Derivation of the modified model

The Fourier's law^31^, is the closest model representing heat conduction, which assumes that there is a linear relationship between the heat flow ${\varvec{q}}$ and the temperature gradient $\nabla \theta$ on the following form:

1 $${\varvec{q}}\left( {{\varvec{x}},t} \right) = - K\nabla \theta \left( {{\varvec{x}},t} \right)$$

The following relations are achieved by the increment in entropy $S$

2 $${\text{div}}{\varvec{q}} + Q = - \rho T_{0} \dot{S}$$

3 $$\rho T_{0} S = \rho C_{e} \theta + \gamma T_{0} {\text{div}}{\varvec{u}}$$

From Eqs. (2) and (3), we obtain the energy equation as

4 $$\rho C_{e} \frac{\partial \theta }{{\partial t}} + \gamma T_{0} \frac{\partial }{\partial t}\left( {{\text{div}}{\varvec{u}}} \right) = - {\text{div}}{\varvec{q}} + Q$$

Among the governing equations in the two-temperature model (2TT), in addition to the energy Eq. (4), the equation that connecting the two temperatures $\theta$ and $\varphi$ is given by^7–12^:

5 $$\theta = \left( {1 - a\nabla^{2} } \right)\varphi$$

which, $a > 0$ is the temperature distinction parameter (two-temperature).

The classical Fourier's law (1) has been replaced, according to Quintanilla^29^ with

6 $${\varvec{q}}\left( {{\varvec{x}},t} \right) = - K\nabla \varphi \left( {{\varvec{x}},t} \right)$$

In the DPL model, the heat Eq. (6) is modified by introducing two phase-lags as^11^:

7 $${\varvec{q}}\left( {{\varvec{x}},t + \tau_{q} } \right) = - K\nabla \varphi \left( {{\varvec{x}},t + \tau_{\varphi } } \right)$$

In the above equation, $\tau_{q}$ and $\tau_{\varphi }$ are respectively the phase lags of the heat flux and conductive temperature gradient.

Introducing the phase lag of the temperature $\tau_{\theta }$, in addition to the phase lag $\tau_{q}$ of conductive temperature, we get the following relation

8 $$\theta \left( {{\varvec{x}},t + \tau_{\theta } } \right) = \left( {1 - a\nabla^{2} } \right)\varphi \left( {{\varvec{x}},t + \tau_{\varphi } } \right)$$

Taylor-series-expansion is applied to both sides of Eqs. (7) and (8) and maintain the terms up to a suitable higher-order of time-deferential ($m,n,p$) in $\tau_{q}$, $\tau_{\varphi }$, and $\tau_{\theta }$ respectively, to acquire

9 $$\left( {1 + \mathop \sum \limits_{r = 1}^{m} \frac{{\tau_{q}^{r} }}{r!}\frac{{\partial^{r} }}{{\partial t^{r} }}} \right){\varvec{q}} = - K\left( {1 + \mathop \sum \limits_{r = 1}^{n} \frac{{\tau_{\varphi }^{r} }}{r!}\frac{{\partial^{r} }}{{\partial t^{r} }}} \right)\nabla \varphi$$

10 $$\left( {1 + \mathop \sum \limits_{r = 1}^{p} \frac{{\tau_{\theta }^{r} }}{r!}\frac{{\partial^{r} }}{{\partial t^{r} }}} \right)\theta = \left( {1 - a\nabla^{2} } \right)\left( {1 + \mathop \sum \limits_{r = 1}^{n} \frac{{\tau_{\varphi }^{r} }}{r!}\frac{{\partial^{r} }}{{\partial t^{r} }}} \right)\varphi$$

It was highlighted that the proposed models associated with the higher-order time-differential have been extensively considered in many papers regarding their thermodynamic consistency and also with regard to well-presented issues and stimulating stability^32–37^. If we adjoin Eq. (9) with the energy Eq. (4), we get

11 $$K\left( {1 + \mathop \sum \limits_{r = 1}^{n} \frac{{\tau_{\varphi }^{r} }}{r!}\frac{{\partial^{r} }}{{\partial t^{r} }}} \right)\nabla^{2} \varphi = \left( {1 + \mathop \sum \limits_{r = 1}^{m} \frac{{\tau_{q}^{r} }}{r!}\frac{{\partial^{r} }}{{\partial t^{r} }}} \right)\left( {\rho C_{e} \dot{\theta } + \gamma T_{0} \dot{e} - \rho Q} \right)$$

By studying a system of equations similar to the foundational equations of type (9) or (10), Chiriţă et al.^35^ explain that there are some restrictions to choosing the higher orders $m,n,$ and $p$, for example when $m \ge 5$ leads to an unstable system, and therefore cannot describe a real physical state.

In addition, the field equations, the constitutive relations and the train–displacement relation to thermoelastic isotropic materials at uniform environmental temperature $T_{0}$ are:

12 $$\sigma_{ij} = 2\mu e_{ij} + \delta_{ij} \left[ {\lambda e_{ij} - \gamma \theta } \right]$$

13 $$2e_{ij} = u_{j,i} + u_{i,j}$$

14 $$\mu u_{i,jj} + \left( {\lambda + \mu } \right)u_{j,ij} - \gamma \theta_{,i} + F_{i} = \rho \ddot{u}_{i}$$

## Application to the model

As to achieve the accuracy of the presented model, we are now studying a thermoelastic body that is exposed to thermal shock and is affected by an external force. It is assumed that all field variables depend only on the distance $x$ and instant time $t$. Then displacement components have the form

15 $$u_{x} = u\left( {x,t} \right),\quad u_{y} = 0,\quad u_{z} = 0.{ }$$

The non-zero strain is given by

16 $$e = \frac{{\partial u\left( {x,t} \right)}}{\partial x}{ }$$

The components of the external body strength can be chosen as

17 $$F_{x} = e^{ - \omega x} ,{ }\quad \omega > 0,\quad F_{y} = 0,\quad F_{z} = 0.{ }$$

Equations (9), (11), (12) and (14) then reduce to

18 $$\left( {\lambda + 2\mu } \right)\frac{{\partial^{2} u}}{{\partial x^{2} }} - \gamma \frac{\partial \theta }{{\partial x}} + \rho e^{ - \omega x} = \rho \frac{{\partial^{2} u}}{{\partial t^{2} }}$$

19 $$K\left( {1 + \mathop \sum \limits_{r = 1}^{n} \frac{{\tau_{\varphi }^{r} }}{r!}\frac{{\partial^{r} }}{{\partial t^{r} }}} \right)\frac{{\partial^{2} \varphi }}{{\partial x^{2} }} = \left( {1 + \mathop \sum \limits_{r = 1}^{m} \frac{{\tau_{q}^{r} }}{r!}\frac{{\partial^{r} }}{{\partial t^{r} }}} \right)\left( {\rho C_{e} \frac{\partial \theta }{{\partial t}} + \gamma T_{0} \frac{{\partial^{2} u}}{\partial t\partial x}} \right)$$

20 $$\left( {1 + \mathop \sum \limits_{r = 1}^{p} \frac{{\tau_{\theta }^{r} }}{r!}\frac{{\partial^{r} }}{{\partial t^{r} }}} \right)\theta = \left( {1 - a\frac{{\partial^{2} }}{{\partial x^{2} }}} \right)\left( {1 + \mathop \sum \limits_{r = 1}^{n} \frac{{\tau_{\varphi }^{r} }}{r!}\frac{{\partial^{r} }}{{\partial t^{r} }}} \right)\varphi$$

21 $$\sigma_{xx} = \sigma = \left( {\lambda + 2\mu } \right)\frac{\partial u}{{\partial x}} - \gamma \theta$$

We will consider the dimensionless quantities:

22 $$\begin{aligned} & \left\{ {x^{\prime } ,u^{\prime } } \right\} = \eta c_{0} \left\{ {x,u} \right\}, \quad \sigma^{\prime } = \frac{\sigma }{\lambda + 2\mu }, \left\{ {t^{\prime } ,\tau_{q}^{\prime } ,\tau_{\theta }^{\prime } ,\tau_{\varphi }^{\prime } } \right\} = c_{0}^{2} \eta \left\{ {t,\tau_{0} ,\tau_{1} ,\tau_{\theta } ,\tau_{q} } \right\}, \\ & a^{\prime } = \eta^{2} c_{0}^{2} a, \theta^{\prime } = \frac{\gamma }{\lambda + 2\mu }\theta , F^{\prime } = \frac{\rho }{{\eta c_{0} \left( {\lambda + 2\mu } \right)}}F, c_{0}^{2} = \frac{{\left( {\lambda + 2\mu } \right)}}{\rho }, \eta = \frac{{\rho C_{e} c_{0}^{2} }}{K}. \\ \end{aligned}$$

The governing Eqs. (18)–(21), by using Eq. (22) may be reformulated in the non-dimensional forms as (neglecting the primes):

23 $$\frac{{\partial^{2} u}}{{\partial x^{2} }} - \frac{\partial \theta }{{\partial x}} + e^{ - \omega x} = \frac{{\partial^{2} u}}{{\partial t^{2} }}$$

24 $$\left( {1 + \mathop \sum \limits_{r = 1}^{n} \frac{{\tau_{\varphi }^{r} }}{r!}\frac{{\partial^{r} }}{{\partial t^{r} }}} \right)\frac{{\partial^{2} \varphi }}{{\partial x^{2} }} = \left( {1 + \mathop \sum \limits_{r = 1}^{m} \frac{{\tau_{q}^{r} }}{r!}\frac{{\partial^{r} }}{{\partial t^{r} }}} \right)\left( {\frac{\partial \theta }{{\partial t}} + \varepsilon \frac{{\partial^{2} u}}{\partial t\partial x}} \right),$$

25 $$\left( {1 + \mathop \sum \limits_{r = 1}^{p} \frac{{\tau_{\theta }^{r} }}{r!}\frac{{\partial^{r} }}{{\partial t^{r} }}} \right)\theta = \left( {1 - a\frac{{\partial^{2} }}{{\partial x^{2} }}} \right)\left( {1 + \mathop \sum \limits_{r = 1}^{n} \frac{{\tau_{\varphi }^{r} }}{r!}\frac{{\partial^{r} }}{{\partial t^{r} }}} \right)\varphi ,$$

26 $$\sigma = \frac{\partial u}{{\partial x}} - \theta$$

where $$\varepsilon = \frac{{\gamma^{2} T_{0} }}{{\rho^{2} c_{0}^{2} C_{e} }}$$.

Homogeneous initial conditions are assumed to be

27 $$\begin{aligned} & \theta \left( {x,0} \right) = \frac{{\partial^{r} \theta \left( {x,0} \right)}}{{\partial t^{r} }} = 0,\quad \varphi \left( {x,0} \right) = \frac{{\partial^{r} \varphi \left( {x,0} \right)}}{{\partial t^{r} }} = 0, \\ & u\left( {x,0} \right) = \frac{{\partial^{r} u\left( {x,0} \right)}}{{\partial t^{r} }} = 0,\quad { }r = \left\{ {m, n, p} \right\}. \\ \end{aligned}$$

From the description of the previous problem, we find that the boundary conditions are in the form

28 $$\begin{aligned} & \sigma \left( {0,t} \right) = 0 \\ & \varphi \left( {0,t} \right) = \varphi_{0} H\left( t \right) \\ \end{aligned}$$

where the parameter $\varphi_{0}$ is constant and $H\left( t \right)$ denotes the Heaviside unit step function.

## Solution of the problem

To get the solution of the problem, we perform the Laplace transform described by

29 $$\overline{f}\left( {x,t} \right) = \mathop \smallint \limits_{0}^{\infty } f\left( {x,t} \right)e^{ - st} dt$$

Transforming Eqs. (23)–(26), we obtain

30 $$s^{2} \overline{u} = {\text{D}}^{2} \overline{u} - {\text{D}}\overline{\theta } + \frac{1}{s}e^{ - \omega x}$$

31 $${ }\ell_{\varphi } {\text{D}}^{2} \overline{\varphi } = \ell_{q} \left( {\overline{\theta } + \varepsilon {\text{D}}\overline{u}} \right),$$

32 $$\ell_{\theta } \overline{\theta } = { }\ell_{\varphi } \left( {1 - a{\text{D}}^{2} } \right)\overline{\varphi },$$

33 $$\begin{array}{*{20}c} {\overline{\sigma } = {\text{D}}\overline{u} - \overline{\theta }} \\ \end{array}$$

where

34 $$\begin{aligned} { } & \ell_{q} = 1 + \mathop \sum \limits_{r = 1}^{m} \frac{{\tau_{q}^{r} }}{r!}s^{r} ,{ }\ell_{\theta } = 1 + \mathop \sum \limits_{r = 1}^{p} \frac{{\tau_{\theta }^{r} }}{r!}s^{r} { } \\ & \ell_{\varphi } = 1 + \mathop \sum \limits_{r = 1}^{n} \frac{{\tau_{\varphi }^{r} }}{r!}s^{r} ,\quad D = \frac{{\text{d}}}{{{\text{d}}x}}.{ } \\ \end{aligned}$$

Eliminating $\overline{\theta }$ from Eqs. (30)–(32), we obtain

35 $$\left( {{\text{D}}^{2} - s^{2} } \right)\overline{e} = \alpha_{1} \left( {{\text{D}}^{2} - a{\text{D}}^{4} } \right)\overline{\varphi } + \frac{\omega }{s}e^{ - \omega x}$$

36 $${ }\left[ {{\text{D}}^{2} - \alpha_{2} } \right]\overline{\varphi } = \alpha_{3} \overline{e}$$

37 $$\begin{array}{*{20}c} {\overline{\sigma } = \overline{e} - \alpha_{1} \left( {1 - a{\text{D}}^{2} } \right)\overline{\varphi }} \\ \end{array}$$

where

38 $$\alpha_{1} = \frac{{\ell_{\varphi } }}{{\ell_{\theta } }},\quad \alpha_{2} = \frac{{\ell_{q} }}{{a\ell_{q} + \ell_{\theta } }},\quad \alpha_{3} = \frac{{\varepsilon \ell_{\theta } \ell_{q} }}{{a\ell_{q} \ell_{\varphi } + \ell_{\varphi } \ell_{\theta } }}.{ }$$

Eliminating $\overline{e}$ between Eqs. (35) and (36), we get

39 $$\left( {{\text{D}}^{4} - A{\text{D}}^{2} + B} \right)\overline{\varphi } = \alpha_{4} e^{ - \omega x}$$

where

40 $${\text{A}} = \frac{{s^{2} + \alpha_{2} + \alpha_{1} \alpha_{3} }}{{1 + a\alpha_{1} \alpha_{3} }},{\text{ B}} = \frac{{s^{2} \alpha_{2} }}{{1 + a\alpha_{1} \alpha_{3} }},{ }\alpha_{4} = { }\frac{{\omega \alpha_{3} }}{{s\left( {1 + a\alpha_{1} \alpha_{3} } \right)}}.{ }$$

The general solution of Eq. (39) which is bounded as $x \to \infty$ is given by

41 $$\overline{\varphi }\left( x \right) = C_{1} {\text{e}}^{{ - m_{1} x}} + C_{2} {\text{e}}^{{ - m_{2} x}} + C_{3} e^{ - \omega x}$$

where $C_{3} = \alpha_{4} /\left( {\omega^{4} - A\omega^{2} + B} \right)$ and $C_{1}$ and $C_{2}$ are some parameters.

Also, the parameters $m_{1}$ and $m_{1}$ are the roots of the equation

42 $$m^{4} - Am^{2} + B = 0$$

Substituting (41) into Eqs. (36) and (32), we obtain

43 $$\overline{e}\left( x \right) = \frac{{\left( {m_{1}^{2} - \alpha_{2} } \right)}}{{\alpha_{3} }}C_{1} {\text{e}}^{{ - m_{1} x}} + \frac{{\left( {m_{2}^{2} - \alpha_{2} } \right)}}{{\alpha_{3} }}C_{2} {\text{e}}^{{ - m_{2} x}} + \frac{{\left( {\omega^{2} - \alpha_{2} } \right)}}{{\alpha_{3} }}C_{3} e^{ - \omega x}$$

44 $$\begin{aligned} \overline{\theta }\left( x \right) & = \alpha_{1} \left( {1 - am_{1}^{2} } \right)C_{1} {\text{e}}^{{ - m_{1} x}} + \alpha_{1} \left( {1 - am_{2}^{2} } \right)C_{2} {\text{e}}^{{ - m_{2} x}} \\ &\quad + \;\alpha_{1} \left( {1 - a\omega^{2} } \right)C_{3} e^{ - \omega x} \\ \end{aligned}$$

Introducing Eq. (43) into Eq. (16), we get

45 $$\overline{u}\left( x \right) = - \frac{{\left( {m_{1}^{2} - \alpha_{2} } \right)}}{{m_{1} \alpha_{3} }}C_{1} {\text{e}}^{{ - m_{1} x}} - \frac{{\left( {m_{2}^{2} - \alpha_{2} } \right)}}{{m_{2} \alpha_{3} }}C_{2} {\text{e}}^{{ - m_{2} x}} - \frac{{\left( {\omega^{2} - \alpha_{2} } \right)}}{{\omega \alpha_{3} }}C_{3} e^{ - \omega x}$$

Using Eqs. (41) and (43) into Eq. (37) we have

46 $$\begin{aligned} \overline{\sigma }\left( x \right) = & \left[ {\frac{{\left( {m_{1}^{2} - \alpha_{2} } \right)}}{{\alpha_{3} }} - \alpha_{1} \left( {1 - am_{1}^{2} } \right)} \right]C_{1} {\text{e}}^{{ - m_{1} x}} \\ & + \left[ {\frac{{\left( {m_{2}^{2} - \alpha_{2} } \right)}}{{\alpha_{3} }} - \alpha_{1} \left( {1 - am_{2}^{2} } \right)} \right]C_{2} {\text{e}}^{{ - m_{2} x}} \\ & + \left[ {\frac{{\left( {\omega^{2} - \alpha_{2} } \right)}}{{\alpha_{3} }} - \alpha_{1} \left( {1 - a\omega^{2} } \right)} \right]C_{3} e^{ - \omega x} \\ \end{aligned}$$

By taking the Laplace transform to the boundary conditions (28), we get

47 $$\begin{array}{*{20}l} {\overline{\sigma } = 0{ }} \hfill & {{\text{on}}\quad x = 0} \hfill \\ {\overline{\varphi } = \varphi_{0} /s} \hfill & {{\text{on}}\quad x = 0} \hfill \\ \end{array}$$

Substituting the functions of $\overline{\sigma }$ and $\overline{\varphi }$ given in (41) and (46) into the boundary conditions (47), we can obtain the integral parameters $C_{1}$ and $C_{2}$.

## Special cases

- The classical coupled thermoelasticity (CTE)^1^ is yielded when $\tau_{q} = \tau_{\theta } = \tau_{\varphi } = 0$, $a = 0$,${ }\theta = \varphi$. In this case, the heat conduction equation can be expressed as
  48 $$K\nabla^{2} \theta = \rho C_{e} \frac{\partial \theta }{{\partial t}} + \gamma T_{0} \frac{\partial e}{{\partial t}} - \rho Q$$
- Lord–Shulman theory (LS)^38^ is given by setting $$\tau_{q} = \tau_{0} > 0,$$$$a = 0$$, $$\theta = \varphi$$, $$\tau_{\theta } ,\tau_{\varphi } \to 0$$ and taken $$m = 1$$. In this case, the heat equation has the form
  49 $$K\nabla^{2} \theta = \left( {1 + \tau_{0} \frac{\partial }{\partial t}} \right)\left( {\rho C_{e} \frac{\partial \theta }{{\partial t}} + \gamma T_{0} \frac{\partial e}{{\partial t}} - \rho Q} \right)$$
- The heat equation proposed by Tzou (DPL)^3,6^ is given when $a = 0$, $\theta = \varphi$, $\tau_{\theta } = \tau_{\varphi }$, $n = 1$, $m = 2$ and take the following the form
  50 $$K\left( {1 + \tau_{\theta } \frac{\partial }{\partial t}} \right)\nabla^{2} \theta = \left( {1 + \tau_{q} \frac{\partial }{\partial t} + \frac{{\tau_{q}^{2} }}{2}\frac{{\partial^{2} }}{{\partial t^{2} }}} \right)\left( {\rho C_{e} \frac{\partial \theta }{{\partial t}} + \gamma T_{0} \frac{\partial e}{{\partial t}} - \rho Q} \right)$$
- Tzou^3^ and Chandrasekharaiah^4^ models with dual-phase-lags (DPL) are obtained by setting $a = 0$, ${ }\theta = \varphi$, $\tau_{\theta } = \tau_{\varphi }$, $m = n = 1$. The heat equation in this case is given by
  51 $$K\left( {1 + \tau_{\theta } \frac{\partial }{\partial t}} \right)\nabla^{2} \theta = \left( {1 + \tau_{q} \frac{\partial }{\partial t}} \right)\left( {\rho C_{e} \frac{\partial \theta }{{\partial t}} + \gamma T_{0} \frac{\partial e}{{\partial t}} - \rho Q} \right)$$
- The two-temperature thermoelasticity model with phase-lags introduced by Mukhopadhyay et al.^21^ (MTTE) is given when we take $a > 0$, $\tau_{\theta } = \tau_{\varphi }$, $m = n = p = 1$. In this case, the two-temperature relation and the heat equation are:
  52 $$\theta = \left( {1 - a\nabla^{2} } \right)\varphi$$
  53 $$K\left( {1 + \tau_{\theta } \frac{\partial }{\partial t}} \right)\nabla^{2} \varphi = \left( {1 + \tau_{q} \frac{\partial }{\partial t}} \right)\left( {\rho C_{e} \frac{\partial \theta }{{\partial t}} + \gamma T_{0} \frac{\partial e}{{\partial t}} - \rho Q} \right)$$
- The governing equations proposed by^20^ (YTTE) can be acquired as special case by taking $$a > 0$$, $$\tau_{\theta } = \tau_{\varphi } = 0$$, $$\tau_{q} = \tau_{0} > 0,$$$$m = n = p = 1$$. In this case
  54 $$\theta = \left( {1 - a\nabla^{2} } \right)\varphi$$
  55 $$K\nabla^{2} \varphi = \left( {1 + \tau_{0} \frac{\partial }{\partial t}} \right)\left( {\rho C_{e} \frac{\partial \theta }{{\partial t}} + \gamma T_{0} \frac{\partial e}{{\partial t}} - \rho Q} \right)$$
- The generalized two-temperature thermoelasticity theory with two-phase-lags and high-order (HTTE) is obained when $a > 0$, $\tau_{q} ,\tau_{\theta } ,\tau_{\varphi } > 0$, $n,m,p \ge 1$.

## Numerical results

In the current section, we will try to provide a practical example to validate the accuracy of the current model. Also, the results can be scheduled to support other researchers to compare their results and verify their accuracy. For the purposes of numerical discussions, we have taken the values of the copper material constants as^39^:

$$\begin{aligned} & C_{E} = 383.1\left( {\frac{{\text{J}}}{{\text{kg K}}}} \right),\quad T_{0} = 293{ }\left( {\text{K}} \right),\quad \alpha_{t} = 1.78 \times 10^{ - 5} { }\left( {\frac{1}{{\text{K}}}} \right),\quad K = 386\left( {\frac{{\text{W}}}{{\text{m K}}}} \right), \\ & \lambda = 7.76 \times 10^{10} { }\left( {\frac{{\text{N}}}{{{\text{m}}^{2} }}} \right),\quad \mu = 3.86 \times 10^{10} { }\left( {\frac{{\text{N}}}{{{\text{m}}^{2} }}} \right),{ }\quad \rho = 8954{ }\left( {\frac{{{\text{kg}}}}{{{\text{m}}^{3} }}} \right). \\ \end{aligned}$$

We performed the calculations when $t = 0.12\,{\text{s}}$, $\varphi_{0} = 1$, and $\varepsilon = 0.0168$. By observing previous literature, most researchers have addressed such a problem without providing tabular results. They provide only some graphical examples to explain and clarify the phenomena.

To obtain the solutions for the distributions of conductive and dynamical temperatures, stress, strain displacement fields in the real domain, we have to employ a numerical inversion technique of the Laplace transform to Eqs. (43)–(46) respectively. Details of these techniques can be found in Honig and Hirdes^40^. In this technique, any function $\overline{g}\left( {x,s} \right)$ in Laplace domain can be inverted to the time domain $g\left( {x,t} \right)$ numerically by the relation

$$g\left( {x,t} \right) = \frac{{e^{\omega t} }}{t}\left( {\frac{1}{2}\overline{g}\left( {x,\omega } \right) + Re\mathop \sum \limits_{n = 1}^{{N_{f} }} \overline{g}\left( {x,\omega + \frac{in\pi }{t}} \right)\left( { - 1} \right)^{n} } \right),$$

where $N_{f}$ is a finite number of terms, $Re$ is the real part and $i$ is imaginary number unit. For faster convergence, numerous numerical experiments have shown that the value of $\omega$ satisfies the relation $\omega t \cong 4.7$^41^. The numerical analysis were performed using the procedure proposed by^40^ with the help of MATHEMATICA programming.

**Figure 1:**
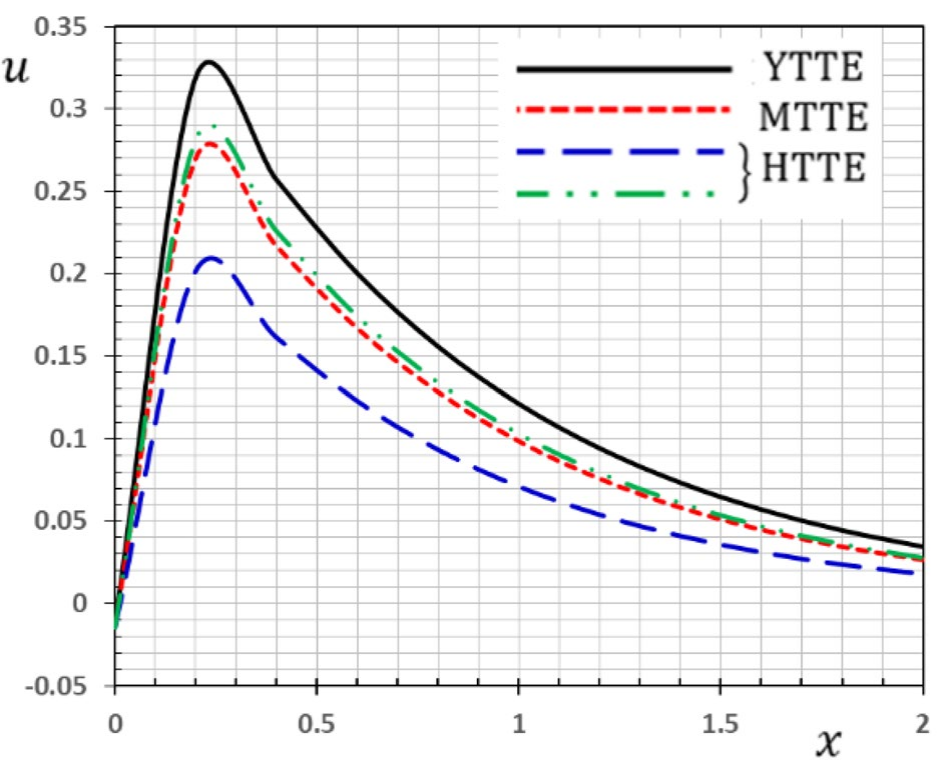
The displacement $u$ for different models of two temperatures.

**Figure 2:**
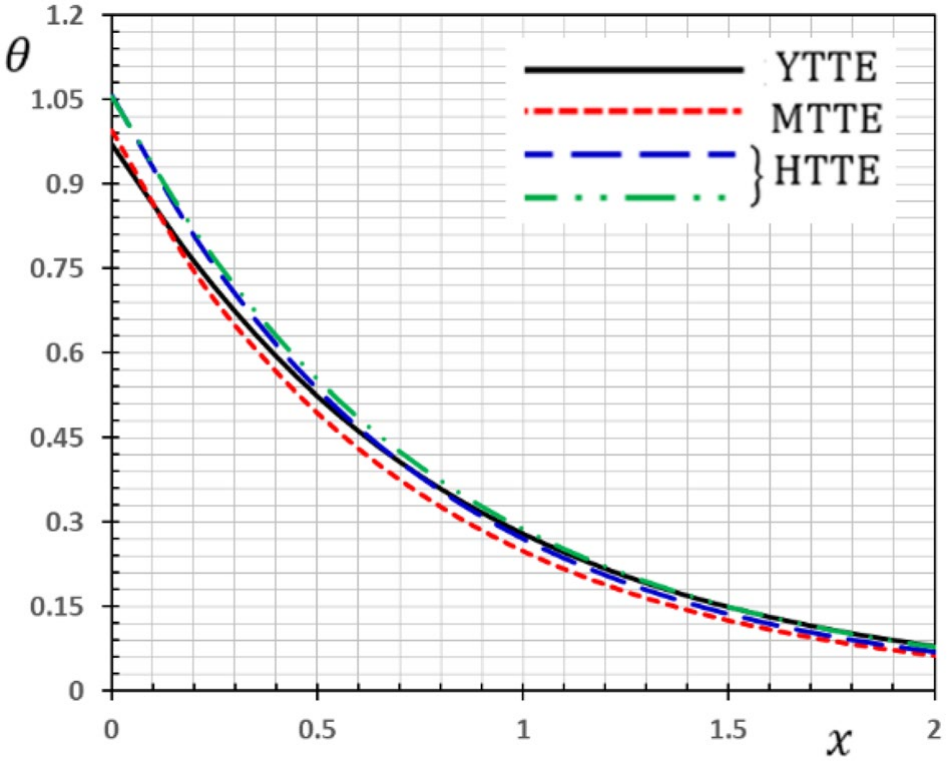
The thermodynamic temperature $\theta$ for different models of two temperatures.

**Figure 3:**
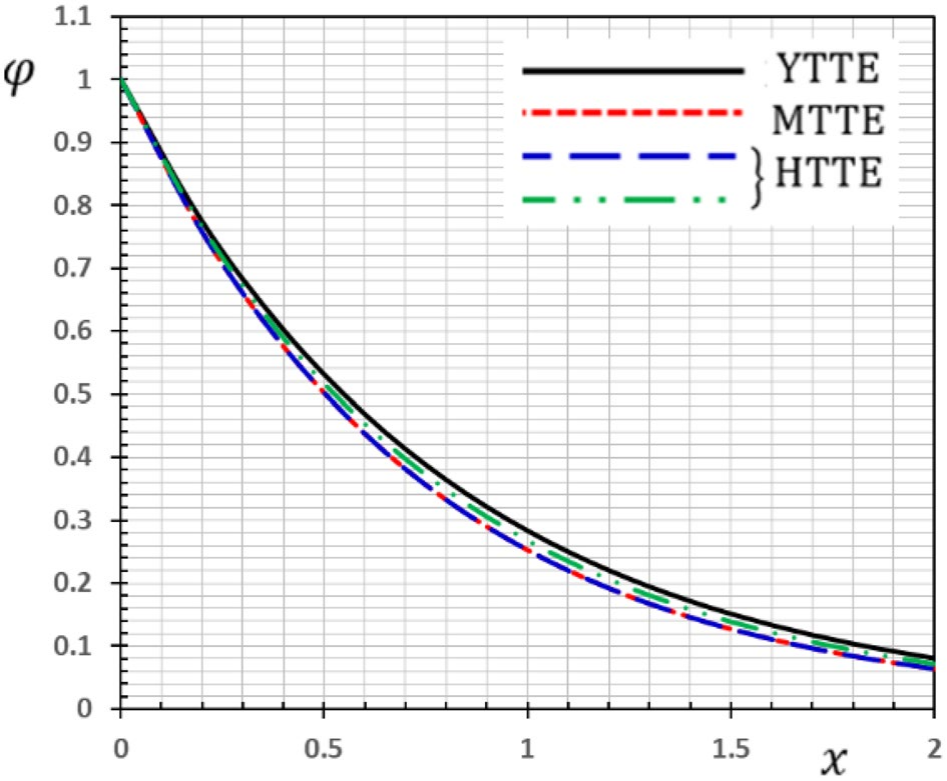
The conductive temperature $\varphi$ for different models of two temperatures.

**Figure 4:**
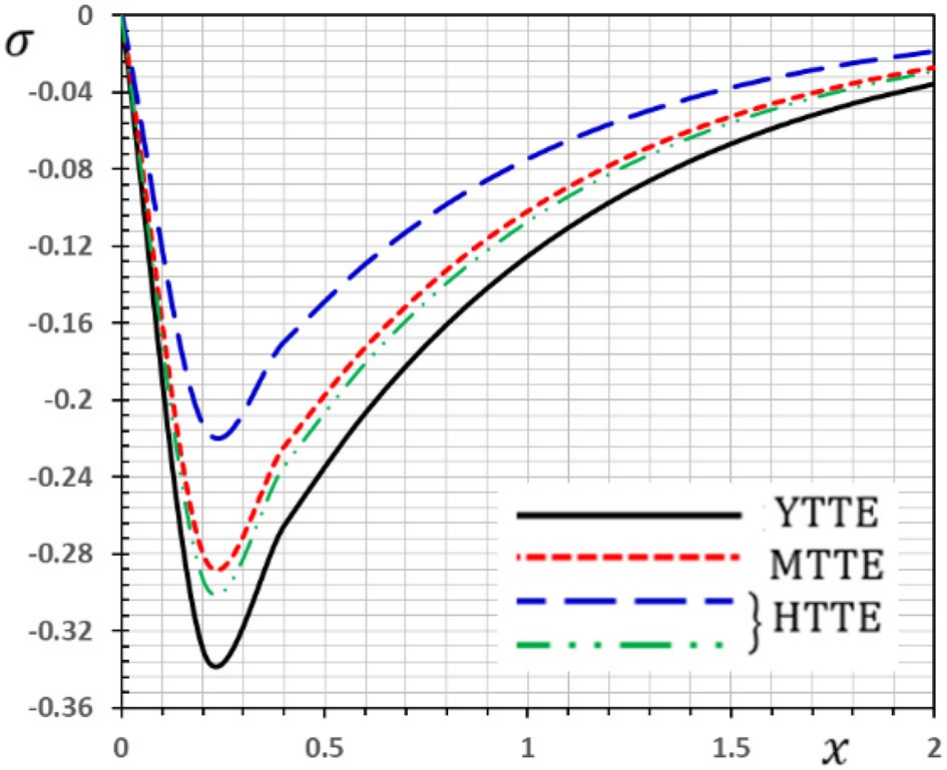
The stress $\sigma$ for different models of two temperatures.

Now we will analyze the effect of higher expansion orders $$m,$$$$n,$$$$p$$ and the temperature discrepancy $$a$$ on the physical variables. To study the influence of the higher-order time-derivatives (HOTD) $$m,$$$$n,$$$$p$$ as well as the distinction parameter of two-temperature $$a$$ on the different fields, we introduce the current numerical results in the form of tables and graphs. Note that if $$a = 0$$ indicates the one-temperature model with HOTD and when $$a = 0.02 \ne 0$$ indicates the two-temperature model with a higher order. The distributions of thermodynamic and conductive temperatures $$\theta$$ and $$\varphi$$, displacement $$u$$ and axial stress $$\sigma$$ are all illustrated in Figs. 1, 2, 3 and 4 and in Tables 1, 2, 3 and 4 for different values of the space *x*. In this section, we compare also the numerical calculations due to the HTTE thermoelastic model to other thermoelasticity models (DPL, LS, YTTE and MTTE).

**Table 1:** Effect of the higher-order time-derivatives on the displacement $u$.

$x$	LS	DPL	YTTE	MTTE	HTTE				
					$m=1$	$m=2$	$m=3$	$m=4$	$m=5$
0.0	− 0.01383	− 0.01383	− 0.01342	− 0.01349	− 0.01462	− 0.01458	− 0.01458	− 0.01458	− 0.01458
0.2	0.33773	0.224234	0.318957	0.201787	0.201735	0.279931	0.281169	0.281293	0.281303
0.4	0.272633	0.179314	0.257051	0.161196	0.161186	0.224568	0.225578	0.225679	0.225688
0.6	0.213114	0.137053	0.200226	0.122816	0.122809	0.17307	0.173882	0.173963	0.17397
0.8	0.166416	0.104538	0.155781	0.093365	0.09336	0.133167	0.133818	0.133883	0.133889
1.0	0.129946	0.07973	0.121197	0.070969	0.070965	0.102457	0.102979	0.103031	0.103036
1.2	0.101469	0.060809	0.094291	0.053945	0.053942	0.078829	0.079247	0.079288	0.079292
1.4	0.079232	0.046378	0.073358	0.041005	0.041002	0.06065	0.060984	0.061017	0.06102
1.6	0.061868	0.035372	0.057072	0.031168	0.031166	0.046663	0.046929	0.046956	0.046958
1.8	0.04831	0.026978	0.044402	0.023692	0.02369	0.035902	0.036114	0.036135	0.036137
2.0	0.037723	0.020575	0.034545	0.018009	0.018007	0.027623	0.027791	0.027808	0.02781

**Table 2:** Effect of the higher-order time-derivatives on thermodynamic temperature $\theta$.

$x$	LS	DPL	YTTE	MTTE	HTTE				
					$m = 1$	$m = 2$	$m = 3$	$m = 4$	$m = 5$
0.0	1.00028	1.00028	0.970667	0.97551	1.05677	1.05423	1.05418	1.05418	1.05418
0.2	0.77677	0.760218	0.762884	0.744943	0.807008	0.817053	0.817222	0.817238	0.81724
0.4	0.606432	0.579712	0.593729	0.566372	0.613558	0.628817	0.629074	0.6291	0.629102
0.6	0.47353	0.442134	0.461924	0.430514	0.466381	0.48381	0.484105	0.484134	0.484137
0.8	0.369756	0.337209	0.359375	0.327242	0.354505	0.372237	0.372539	0.372569	0.372572
1.0	0.288724	0.257184	0.279592	0.248743	0.269465	0.286394	0.286684	0.286713	0.286716
1.2	0.225451	0.19615	0.217522	0.189074	0.204825	0.220348	0.220615	0.220642	0.220644
1.4	0.176043	0.149601	0.169231	0.143719	0.155691	0.169533	0.169773	0.169797	0.169799
1.6	0.137464	0.114098	0.131661	0.109243	0.118343	0.130436	0.130647	0.130668	0.13067
1.8	0.107339	0.087021	0.102432	0.083038	0.089955	0.100356	0.100538	0.100557	0.100558
2.0	0.083815	0.066369	0.079691	0.063119	0.068376	0.077213	0.077369	0.077384	0.077385

**Table 3:** Effect of the higher order Taylor expansions on the conductive temperature $\varphi$,

$x$	LS	DPL	YTTE	MTTE	HTTE				
					$m = 1$	$m = 2$	$m = 3$	$m = 4$	$m = 5$
0.0	1.00028	1.00028	1.00028	1.00028	1.00028	1.00028	1.00028	1.00028	1.00028
0.2	0.77677	0.760218	0.774147	0.757909	0.757899	0.766281	0.766422	0.766436	0.766437
0.4	0.606432	0.579712	0.602173	0.576012	0.576002	0.589462	0.589688	0.589711	0.589713
0.6	0.47353	0.442134	0.468485	0.437834	0.437826	0.453521	0.453786	0.453813	0.453815
0.8	0.369756	0.337209	0.364479	0.332806	0.332799	0.348933	0.349207	0.349235	0.349237
1.0	0.288724	0.257184	0.283563	0.252972	0.252966	0.268464	0.26873	0.268756	0.268758
1.2	0.225451	0.19615	0.220611	0.192289	0.192284	0.206553	0.206799	0.206823	0.206825
1.4	0.176043	0.149601	0.171634	0.146162	0.146158	0.158919	0.15914	0.159162	0.159164
1.6	0.137464	0.114098	0.133531	0.111101	0.111097	0.12227	0.122465	0.122484	0.122486
1.8	0.107339	0.087021	0.103886	0.08445	0.084447	0.094073	0.094242	0.094259	0.09426
2.0	0.083815	0.066369	0.080823	0.064192	0.06419	0.072379	0.072523	0.072537	0.072539

**Table 4:** Effect of the higher order Taylor expansions on the stress $\sigma$.

$x$	LS	DPL	YTTE	MTTE	HTTE				
					$m = 1$	$m = 2$	$m = 3$	$m = 4$	$m = 5$
0.0	0	0	0	0	0	0	0	0	0
0.2	− 0.34847	− 0.23475	− 0.32951	− 0.21209	− 0.2129	− 0.29123	− 0.29247	− 0.2926	− 0.29261
0.4	− 0.28102	− 0.18733	− 0.26526	− 0.16903	− 0.16967	− 0.23326	− 0.23428	− 0.23438	− 0.23439
0.6	− 0.21966	− 0.14317	− 0.20661	− 0.12877	− 0.12926	− 0.17976	− 0.18058	− 0.18066	− 0.18067
0.8	− 0.17153	− 0.1092	− 0.16075	− 0.09789	− 0.09826	− 0.13832	− 0.13897	− 0.13904	− 0.13904
1.0	− 0.13394	− 0.08329	− 0.12506	− 0.07441	− 0.07469	− 0.10642	− 0.10694	− 0.107	− 0.107
1.2	− 0.10459	− 0.06352	− 0.0973	− 0.05656	− 0.05677	− 0.08188	− 0.0823	− 0.08234	− 0.08234
1.4	− 0.08167	− 0.04845	− 0.0757	− 0.04299	− 0.04316	− 0.06299	− 0.06333	− 0.06337	− 0.06337
1.6	− 0.06377	− 0.03695	− 0.05889	− 0.03268	− 0.0328	− 0.04847	− 0.04874	− 0.04876	− 0.04877
1.8	− 0.04979	− 0.02818	− 0.04582	− 0.02484	− 0.02493	− 0.03729	− 0.0375	− 0.03753	− 0.03753
2.0	− 0.03888	− 0.02149	− 0.03565	− 0.01888	− 0.01895	− 0.02869	− 0.02886	− 0.02888	− 0.02888

The phenomenon of limited wave propagation speeds is observed from tables and figures. Also, it is evident from tables and figures that all models display distinctly different values near the surface boundaries, and the differences decrease with increasing distance, due to the effect of thermal shock applied to the stress-free boundary. The conductive temperature profile takes the maximum value at the surface $x = 0$ (thermal shock) and then gradually ultimately decreases to zero. It is detected that thermal stress $\sigma$ disappears on the surface $x = 0$, which meets the state of the mechanical condition of the problem given in the Eq. (47). In Tables 1, 2, 3 and 4, various terms have been considered of the modified heat Eq. (24) and also in the equation related to the conductive and thermodynamic temperatures (25).

It can be clearly observed from the tables that the HTTE thermoelastic model gives perfect results for all HOTD parameters ($m,n,p$). As appeared on Tables 1, 2, 3 and 4, it is enough to put $m = 4,{ }n = 2,{ }p = 1$ for very accurate and close numerical results. Besides the first and second-order approximation, higher-order approximations, leading to higher-order DPL models, were also considered in the literature^42^.

Wang et al.^43^ investigated a well-posedness problem, given some suitable restrictions on the parameters of phase lags $\tau_{q} ,\tau_{\theta }$ and $\tau_{\varphi }$. Quintanilla and Racke^44^ observed that there an area of influence result when $m = n$, however when $m = n + 1$ they have founded some spatial approximations explaining the Phragmén–Lindelöf alternative. It is worth noting that our choices of the HOTD parameters $\left( {m,n,p} \right)$ are capable to involve several models of heat conduction: when we take $m = n$, we have a diffusive behavior; but by taking $m = n + 1$, we get a wavelike behavior^6,30^.

Also, we have found that the HOTD parameters $\left( {m,n,p} \right)$ and the two-temperature parameter $a$ have a distinguished influence on the studied fields. Consequently, according to the results, it is significant to separate between the thermodynamic and the conductive temperatures. The presence of the high-order parameters increases the magnitude of the strain. The HTTE and MTTE models are largely closed to one another while the YTTE and LS models are closed to each other.

Table 1 discusses the effect of the HOTD parameters $$\left( {m,n,p} \right)$$ on the displacement $$u$$ for different models of thermoelasticity. It is clear from the table that the HTTE model with ($$m = n = p = 1$$) gives the smallest values of the displacement, while the LS theory gives the largest displacement values when we take the parameters $$\tau_{q} = \tau_{0} > 0,$$$$a = 0$$, $$\tau_{\theta } ,\tau_{\varphi } \to 0$$, $$\theta = \varphi$$ and $$m = 1$$.

The variations of the displacement $u$ versus distance $x$ for the YTTE, MTTE and HTTE models are illustrated in Fig. 1. It can be seen that the displacement beginning at $x = 0$ with the minimum values for all the models and increases with $x$ to achieve the maximum value at $x = 0.4$. After that, the values of the displacement $u$ drop quickly with distance.

The effect of the HOTD parameters $\left( {m,n,p} \right)$ on the distributions of thermodynamic temperature $\theta$ and conductive temperature $\varphi$ of the medium are displayed in Tables 2 and 3 as well as Figs. 2 and 3 for different thermoelasticity theories when the two-temperature parameter $a$ is present or absent.

From the Tables and figures, we can see that the influences of the HOTD parameters on the fields $\theta$ and $\varphi$ are very notable. Also, the parameter $a$ has a fundamental role in varying the values of the studied fields. It is noted that from Tables and Figs. 2 and 3, the variations of the fields $\theta$ and $\varphi$ is qualitatively similar for all different thermoelastic models. Moreover, it is observed that the conductive and thermodynamic temperatures increase when the high-order approximations parameters decrease. Also, the HTTE model displays the temperature field values compared to the LS, DPL, YTTE and MTTE models.

**Figure 5:**
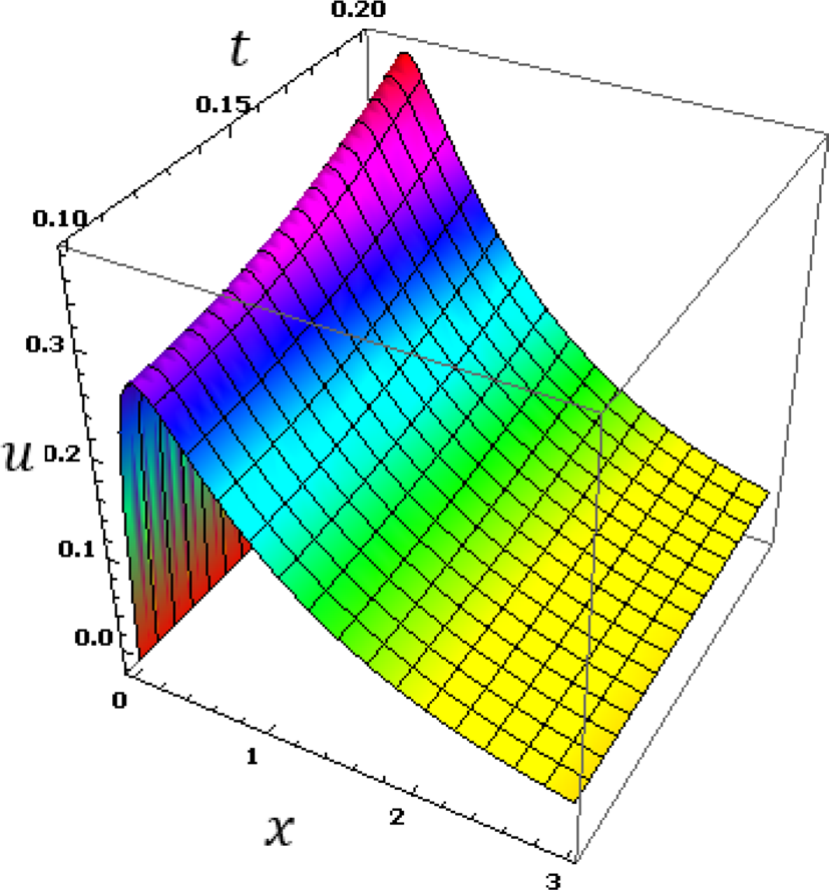
The displacement $u$ with different time instant $t$ and distance $x$.

In the last set of 3D graphs (5–8), the numerical results of studied variables are introduced along the axial axis ($0.0\le x\le 3.0$) and for different time ($0.0\le t\le 0.2$) under the HTTE model. The HTTE theory with $m=4, n=3, p=2$ is used in all the 3D figures. Figure 5 displays the variation of the displacement profile $n$ against the axial distance due to the presence of time effects. The displacement profile grows to reach its maximum value and then decomposes as we move away from the boundary.

**Figure 6:**
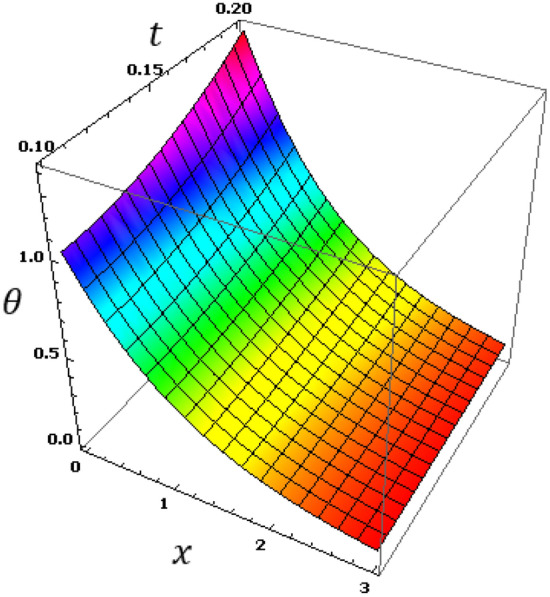
The thermodynamic temperature $\theta$ with different time $t$ and distance $x$.

**Figure 7:**
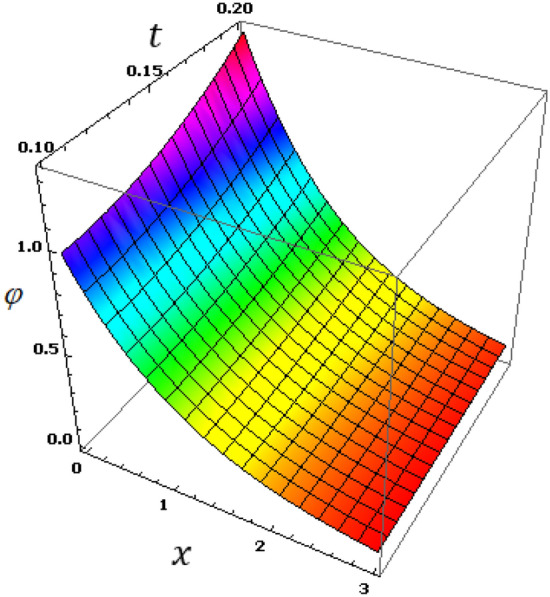
The conductive temperature $\varphi$ with different time instance $t$ and distance $x$.

**Figure 8:**
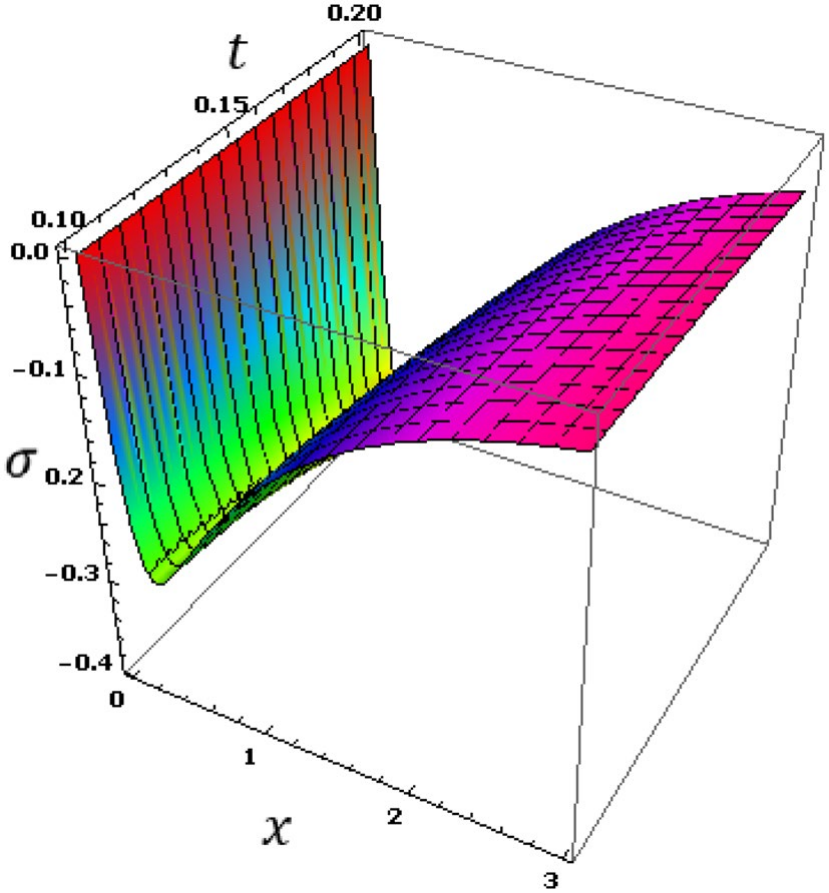
The stress $\sigma$ with different time instance $t$ and distance $x$.

Observing the various results, we find that due to the presence of time, it was observed that the profiles of displacement, conductive and thermodynamic temperatures fields increase with time, which supports the physical reality. A general observation from all these numerical results and graphs (5–8), it is noted that all physical fields are sensitive to the time that is included. Figure 8 shows that the thermal stress field has an equal starting point with a value of zero, which indicates that the mechanical boundary condition is satisfied. Figure 6 displays the conductive temperature variance with distance $x$ and time $t$. From Fig. 7, we can see that all the profiles of $\varphi$ have a coincident beginning point with value $\varphi =1$, which satisfies the thermal boundary conditions (Fig. 8).

## Conclusions

In the current paper, a modified two-temperature thermoelasticity model with higher-order-time derivatives (HOTD) and three different phase-lags has been constructed. The derived model was established by taking into account the Taylor series expansions of Fourier’s heat conduction and the relation for the two temperatures and keeping terms up to appropriate higher-orders in the phase-lags ${\tau }_{q}$, ${\tau }_{\varphi }$ and ${\tau }_{\theta }$. Also, the two temperature model with one relaxation time (YTTE) and two temperature theory with two phase-lags (MTTE) are compared with the higher-order model with two-temperature and three-phase-lags.

To validate the proposed model and show that it is more accurate, the results are tabulated. From the Tables and figures, we observed that the effects of the HOTD parameters on $\theta$ and $\varphi$ fields are very significant. The sensitivity of the physical fields to the variation of the high-order parameters is investigated. For the current HTTE model, $m=4$ is sufficient to obtain valid and effective results.

It is also evident from the results that when the HOTD parameters are lower than or equal to four, the relevant model can be thermodynamically compatible, providing that it makes suitable appropriate assumptions upon the delay times.

This result is consistent with the results got by Chiriţă^25,31^. Finally, this work describes that the theory of thermoelasticity with two-temperature and three-phase-lags and HOTD parameters explains the behavior of the particles of the thermoelastic body more realistically than the model of thermoelasticity with two-temperature with one or two phase-lags. Also, the current study helps some researchers to show how they choose the values of these parameters.

## List of symbols

$$\lambda ,{ }\mu$$: Lamé’s constants
$\alpha_{t}$: Thermal expansion coefficient
$$C_{e}$$: Specific heat
$\gamma = \left( {3\lambda + 2\mu } \right)\alpha_{t}$: Thermal coupling parameter
$$T_{0}$$: Environmental temperature
$$\theta = T - T_{0}$$: Temperature increment
*T*: Absolute temperature
$${\varvec{u}}$$: Displacement vector
$$e = {\text{div}}{\mathbf{u}}$$: Cubical dilatation
$$\sigma_{ij}$$: Stress tensor
$$e_{ij}$$: Strain tensor
${\varvec{q}}$: Heat flux vector
$$K$$: Thermal conductivity
$$\rho$$: Material density
$$Q$$: Heat source
$$t$$: The time
$\delta_{ij}$: Kronecker’s delta function
$$\varphi$$: Conductive temperature
$\tau_{q}$: Phase lag of heat flux
$\tau_{\theta }$: Phase lag of temperature
$\tau_{\varphi }$: Phase lag of ${ }\nabla \varphi$
$\tau_{0}$: Thermal relaxation time
$$m,n,p$$: Higher orders
$$S$$: The entropy
